# Myeloid-Derived Suppressor Cells and CD68^+^CD163^+^M2-Like Macrophages as Therapeutic Response Biomarkers Are Associated with Plasma Inflammatory Cytokines: A Preliminary Study for Non-Small Cell Lung Cancer Patients in Radiotherapy

**DOI:** 10.1155/2022/3621496

**Published:** 2022-07-26

**Authors:** Minghe Lv, Xibing Zhuang, Shali Shao, Xuan Li, Yunfeng Cheng, Duojiao Wu, Xiangdong Wang, Tiankui Qiao

**Affiliations:** Center for Tumor Diagnosis and Therapy, Jinshan Hospital, Fudan University, Shanghai 201508, China

## Abstract

**Background:**

Recent studies show that myeloid-derived suppressor cells (MDSCs) and M2-like macrophages are involved in the treatment of tumors; however, their therapeutic response role is rarely known in non-small cell lung cancer (NSCLC) during radiotherapy. We aim to explore the dynamic alteration of the circulating MDSCs and M2-like macrophages, to examine their relationship, and to evaluate their therapeutic response value for NSCLC patients in radiotherapy.

**Methods:**

Peripheral blood mononuclear cells from healthy controls and NSCLC patients with different radiotherapy phases were isolated to examine the circulating MDSCs and M2-like macrophages by flow cytometry. 40 plasma inflammatory cytokines were measured by multiplex ELISA.

**Results:**

In comparison with healthy controls, the percentages of MDSCs and CD68^+^CD163^+^M2-like macrophages of NSCLC patients were significantly elevated and were distinctly higher in radiotherapy than in preradiotherapy. MDSCs were correlated positively with CD68^+^CD163^+^M2-like macrophages in NSCLC patients in radiotherapy and postradiotherapy. Especially, we found that in comparison with those in the poor group, the percentages of two cells in the good response group were markedly increased during radiotherapy and they had a significantly positive correlation. During radiotherapy, the proportions of MDSCs were clearly increased in adenocarcinoma patients and the percentages of CD68^+^CD163^+^M2-like macrophages were markedly elevated in squamous carcinoma patients. We found that after radiotherapy, the expressions of eotaxin, MIP-1*β*, MCP-1, and BLC were significantly increased in NSCLC patients. Further results showed that the low levels of eotaxin and TNF RII expression before radiotherapy could predict a good therapeutic response. IL-1ra and MIP-1*β* had a positive relation with MDSCs or CD68^+^CD163^+^M2-like macrophages in NSCLC patients during radiotherapy, and eotaxin was correlated with CD68^+^CD163^+^M2-like macrophages but not MDSCs in NSCLC patients after radiotherapy.

**Conclusions:**

MDSCs and CD68^+^CD163^+^M2-like macrophages serve as therapeutic response biomarkers and are associated with the expressions of plasma inflammatory cytokines for NSCLC patients during radiotherapy.

## 1. Background

Lung cancer is the major cause of cancer-related death among males in both more and less developed countries [1] and has surpassed breast cancer as the leading cause of cancer death among females in more developed countries [2]. Non-small cell lung cancer (NSCLC) as the most common type of lung cancer accounts for approximately 85% of all cases [3, 4]. Many NSCLC patients have already developed to the advanced stage when they are diagnosed, and then, they have little chance to accept surgical treatment. Local radiotherapy is a traditional approach to treating terminal NSCLC [5], which can retard the deterioration of primary or secondary lesion and relieve pain by activating the body immune system [6, 7]. However, the correlation between local radiotherapy and the variation of the immune system of cancer patients has been rarely reported. Therefore, it is crucial to carry out further studies on their potential relations and mechanisms, especially the effects of immunosuppressive cells on cancer patients' prognosis in radiotherapy (RT).

Myeloid-derived suppressor cells (MDSCs), a subset of bone marrow-derived immature myeloid cells and one of the major factors negatively regulating immune responses [8], have been confirmed to suppress the activation of T cells [9] and the differentiation of dendritic cells (DCs) [10]. According to the recent research findings, tumor-secreted and host-secreted factors, many of which are proinflammatory molecules, can induce the accumulation of MDSCs [11]. Increased MDSCs have been implicated in the pathogenesis of cancer, which could also serve as a prognostic marker for treatment responses in cancer [12, 13]. Macrophages, a group of terminally differentiated myeloid cells derived from monocytes in peripheral blood, are also crucial drivers to promote tumor inflammation [14]. M2 macrophages that are predominantly characterized as CD68^+^ (pan macrophage marker) and CD163^+^ (M2 specific marker) could dampen the immune responses and limit inflammation reactions. Ionizing radiation (IR) promotes the release of danger signals and chemokines that recruit inflammatory cells into the tumor microenvironment, including antigen-presenting cells that activate cytotoxic T cell function [15]. However, whether the alterations of MDSCs and M2 macrophages of NSCLC patients paly a potential role in improving sensitivities and outcomes in radiotherapy has not yet been investigated in NSCLC patients.

The aim of this study is at analyzing the dynamic alterations of circulating MDSCs and M2-like macrophages of NSCLC patients in radiotherapy, at examining their relationships, and at evaluating the potential connections of plasm cytokines, thereby providing a novel insight as well as therapeutic response factors for NSCLC patients in radiotherapy.

## 2. Materials and Methods

### 2.1. Patients and Controls

From June 2018 to April 2019, peripheral blood was obtained from sixteen NSCLC patients before radiotherapy (preradiotherapy (pre-RT)), during radiotherapy (RT), and after radiotherapy (postradiotherapy (post-RT)). The single radiation dose was 200 cGy. According to the eighth edition of the TNM Classification of Lung Cancer [16], all NSCLC patients (over 44 years old) were diagnosed with inoperable, locally advanced (stage III), or metastatic (stage IV) disease. The evaluation of target lesions in RECIST criteria [17] was as follows: complete response (CR)—the disappearance of all target lesions; partial response (PR)—at least a 30% decrease in the sum of the longest diameter of target lesions, taking the baseline sum longest diameter as reference; progressive disease (PD)—at least a 20% increase in the sum of the longest diameter of target lesions, taking the smallest sum longest diameter recorded since the initial treatment or the appearance of one or more new lesions as reference; and stable disease (SD)—neither sufficient shrinkage to qualify for partial response nor sufficient increase to qualify for progressive disease, taking the smallest sum longest diameter since the initial treatment as reference. We defined CR and PR as the good response group and SD and PD as the poor response group. For controls, blood samples were collected from 8 healthy volunteers (4 males and 4 females; age 56 ± 7 years). All patients provided a written informed consent, and the study was approved by the ethics committee of Jinshan Hospital affiliated to Fudan University, and the ethical code is IEC-2020-S01.

### 2.2. Isolation of Peripheral Blood Mononuclear Cells

EDTA-anticoagulated venous blood samples were freshly collected from healthy controls and NSCLC patients. According to the manufacturer's manual and density-gradient centrifugation, peripheral blood mononuclear cells (PBMCs) were isolated by using human lymphocyte separation medium (Haoyang Biological Products Technology, Tianjin, China). The detached PBMCs were resuspended in cell freezing medium (10% DMSO in fetal bovine serum) and then stored at −80°C.

### 2.3. Flow Cytometry Analysis

The stored PBMCs of healthy controls and NSCLC patients were thawed by water with 37°C in water bath pot. Then, 100 *μ*l of cell suspensions was placed in a flow tube and was resuspend by 4 ml of phosphate buffered solution. The cellular mixture was centrifuged for 5 minutes at 1500 rpm and the supernatant was discarded. M2-like macrophages needed to be fixed and broken and then were intracellularly stained with CD68 (Y1/82A; BD Biosciences) and CD163 (GHI/61; BD Biosciences) for 30 minutes at room temperature, while MDSCs were not. MDSCs were surfacely stained with APC-conjugated anti-human CD11b (ICRF44; BD Biosciences, San Diego, CA), FITC-conjugated anti-human CD33 (HIM3-4; BD Biosciences), and PerCP-Cy5.5-conjugated anti-human HLA-DR^−^ (G46-6; BD Biosciences) for 30 minutes at 4°C. Finally, all samples were resuspend by 200 *μ*l of PBS and were analyzed by using flow cytometry (BD Biosciences).

### 2.4. Plasma Inflammation Cytokine Array

Cytokine profiles were measured by Quantibody Human Inflammation Array 3 (Ray Biotech, Norcross, GA) that detected 40 inflammation-associated cytokines simultaneously by using plasma samples (8 healthy controls and 16 NSCLC patients in pre-RT, RT, and post-RT) which were cryopreserved at −80°C. According to the protocol provided by the manufacturer, the array slides were incubated with thawed plasma samples and then were washed and incubated with a cocktail of biotinylated antibodies. The slides bounded with biotin were then incubated with streptavidin-conjugated Hylite Plus 555 fluor. Relative fluorescent strength was measured by LuxScan 10 K-A Microarray Scanner (CapitalBio Corporation, Beijing, China).

### 2.5. Statistical Analysis

Statistical analysis was performed by using GraphPad Prism version 7.0 (GraphPad Institute Inc, USA), Microsoft Office Excel 2016 (Microsoft, USA), and IBM SPSS Statistics 20 (SPSS Company, USA). One-Way ANOVA and Tukey's test were applied to continuous variables between groups. Statistical significance (*p* < 0.05) was determined by two-tailed Student's *t*-test in two independent groups. The correlation between MDSCs and M2-like macrophages was accessed by Pearson's correlation coefficient.

## 3. Results

### 3.1. Clinical Characteristics of Healthy Controls and NSCLC Patients

In the present study, nine NSCLC patients have a good therapeutic response after radiotherapy, while 7 NSCLC patients have a poor therapeutic response. There was a distant metastasis (stage IV) in twelve patients from sixteen NSCLC patients which included ten adenocarcinoma and six squamous carcinoma patients. Seven lung cancer patients received radiation to the lungs, five to the brain, and four to the bone. Clinical characteristics of NSCLC patients were presented in Table 1. The median age of eight heathy controls (4 females and 4 males) was 54.5 (ranging from 49 to 62).

### 3.2. The Percentages of Peripheral MDSCs and CD68^+^CD163^+^M2-Like Macrophages Were Significantly Elevated in NSCLC Patients

**Figure 1:**
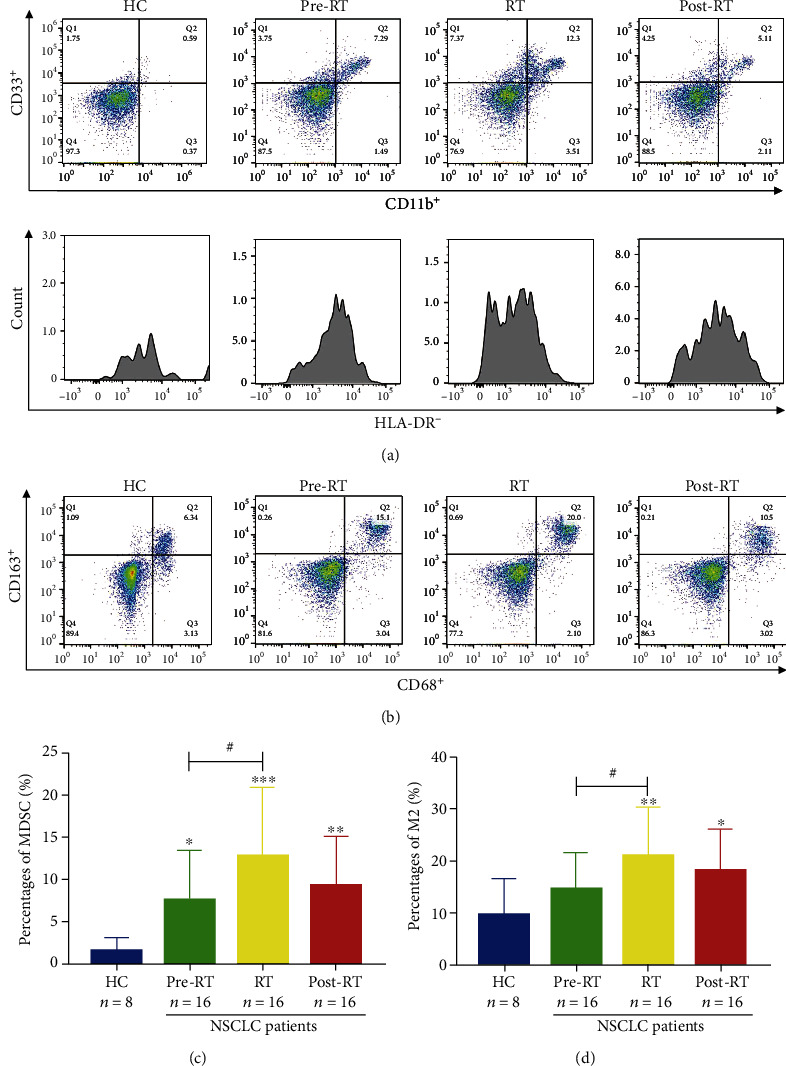
Peripheral MDSCs and CD68^+^CD163^+^M2-like macrophages in HC and NSCLC patients. (a, b) Representative dot plots and peak plots of CD11b^+^CD33^+^HLA-DR^−^ MDSCs and CD68^+^CD163^+^M2-like macrophages in HC and NSCLC patients. (c, d) The percentages of peripheral MDSCs and CD68^+^CD163^+^M2-like macrophages were significantly elevated in NSCLC patients. ^∗^*p* < 0.05, ^∗∗^*p* < 0.01, and ^∗∗∗^*p* < 0.001, compared to HC in two-tailed Student's *t*-test. ^#^*p* < 0.05. The bars denote mean ± SD.

The percentages of MDSCs and CD68^+^CD163^+^M2-like macrophages were analyzed by flow cytometry in healthy controls (HC) and NSCLC patients (Figures 1(a) and 1(b), Figure S1). We found that the percentages of peripheral MDSCs and CD68^+^CD163^+^M2-like macrophages were significantly elevated in NSCLC patients of the pre-RT and RT groups compared to the HC group, while the proportions of MDSCs and CD68^+^CD163^+^M2-like macrophages were decreased in NSCLC patients of the post-RT group, in comparison with the RT group. Otherwise, there were obviously more peripheral MDSCs and CD68^+^CD163^+^M2-like macrophages in NSCLC patients of the RT group than the pre-RT group (Figures 1(c) and 1(d)).

### 3.3. MDSCs Correlated Positively with CD68^+^CD163^+^M2-Like Macrophages in NSCLC Patients

**Figure 2:**
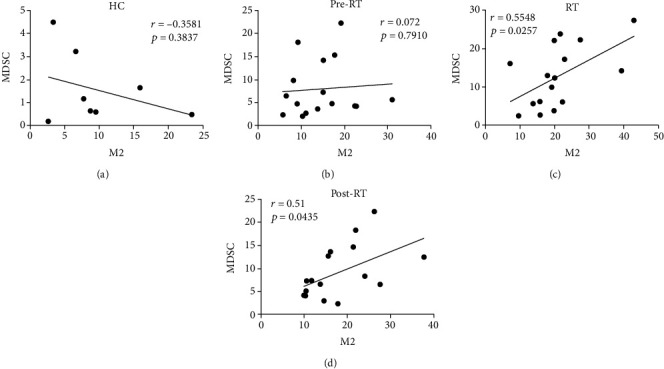
The correlation between peripheral MDSCs and CD68^+^CD163^+^M2-like macrophages in HC and NSCLC patients. (a) The relationship between peripheral MDSCs and CD68^+^CD163^+^M2-like macrophages in HC. The correlation between MDSCs and CD68^+^CD163^+^M2-like macrophages in NSCLC patients among (b) pre-RT, (c) RT, and (d) post-RT group. Data were analyzed by Pearson's correlation test.

In HC, we found that the percentages of MDSCs correlated negatively to CD68^+^CD163^+^M2-like macrophages but their correlation was not significant (Figure 2(a)). Interestingly, the correlation between the proportions of MDSCs and CD68^+^CD163^+^M2-like macrophages was not significant in NSCLC patients of the pre-RT group (*r* = 0.072, *p* = 0.791) (Figure 2(b)), but the percentages of peripheral MDSCs correlated positively to CD68^+^CD163^+^M2-like macrophages in NSCLC patients of the RT group (*r* = 0.5548, *p* = 0.0257) and the post-RT group (*r* = 0.51, *p* = 0.0435) (Figures 2(c) and 2(d)). These data indicated that ionizing radiation (IR) could cause the expansion of peripheric MDSCs and CD68^+^CD163^+^M2-like macrophages in NSCLC patients.

### 3.4. The Value of MDSCs and CD68^+^CD163^+^M2-Like Macrophages of NSCLC Patients in Various Clinical Characteristics

**Figure 3:**
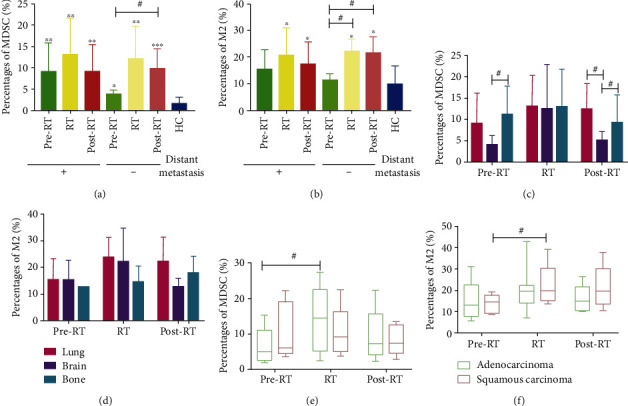
The percentages of MDSCs and CD68^+^CD163^+^M2-like macrophages of NSCLC patients in various clinical characteristics. (a, b) The proportions of MDSCs and CD68^+^CD163^+^M2-like macrophages in NSCLC patients with or without metastasis. (c, d) The percentages of MDSCs and CD68^+^CD163^+^M2-like macrophages in NSCLC patients with a different radiotherapy area. (e, f) The proportions of MDSCs and CD68^+^CD163^+^M2-like macrophages in NSCLC patients with diverse pathologic types. ^∗^*p* < 0.05, ^∗∗^*p* < 0.01, and ^∗∗∗^*p* < 0.001, compared to the HC group in two-tailed Student's *t*-test. ^#^*p* < 0.05. The bars denote mean ± SD.

To further investigate the value of MDSCs and CD68^+^CD163^+^M2-like macrophages of NSCLC patients in the pre-RT, RT, and post-RT groups, we reviewed and classified the clinical data of these patients. In this study, we found that the percentages of MDSCs as well as CD68^+^CD163^+^M2-like macrophages of NSCLC patients in RT and post-RT were elevated in NSCLC patients without metastasis but were not significantly increased in NSCLC patients with metastasis. Furthermore, there were higher percentages of MDSCs of NSCLC patients with metastasis in pre-RT than NSCLC patients without metastasis in pre-RT (Figures 3(a) and 3(b)). The selection of the radiotherapy area could affect the response to radiotherapy. As shown in Figures 3(c) and 3(d), the percentages of MDSCs and CD68^+^CD163^+^M2-like macrophages did not show clear difference in NSCLC patients with lung, brain or bone radiation, but after radiotherapy, the percentages of MDSCs in NSCLC patients with brain radiation were significantly less than NSCLC patients with lung and bone radiation. Hence, we speculated that the alteration of the percentages of MDSCs of NSCLC patients after radiotherapy was correlated with the radiotherapy area. In addition, we analyzed the percentages of MDSCs and CD68^+^CD163^+^M2-like macrophages in non-small cell lung squamous carcinoma and lung adenocarcinoma patients, finding that during radiotherapy, the proportions of MDSCs were markedly increased in non-small cell lung adenocarcinoma patients but not in non-small cell lung squamous carcinoma patients; however, the proportions of CD68^+^CD163^+^M2-like macrophages were markedly elevated in non-small cell lung squamous carcinoma patients but not in non-small cell lung adenocarcinoma patients (Figures 3(e) and 3(f)).

### 3.5. The ROC Curve Analysis about MDSCs and CD68^+^CD163^+^M2-Like Macrophages in NSCLC Diagnosis and Radiotherapy

**Figure 4:**
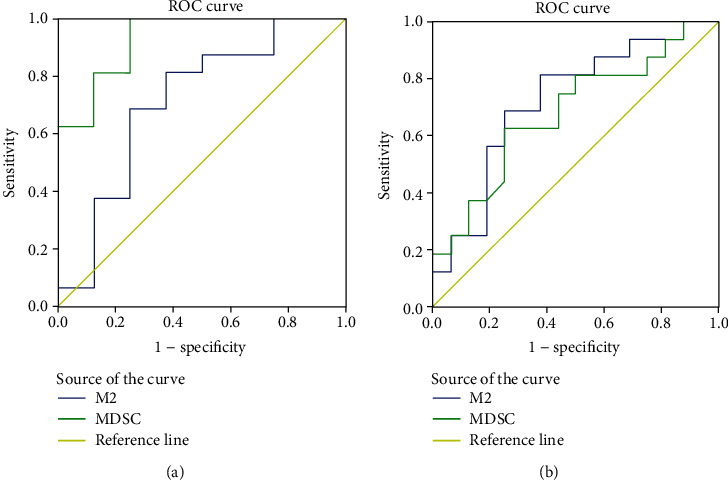
The ROC curve analysis about MDSCs and CD68^+^CD163^+^M2-like macrophages in NSCLC diagnosis and radiotherapy. (a) The ROC curve analysis in NSCLC diagnosis. (b) The ROC curve analysis in NSCLC radiotherapy.

To examine the efficiency of MDSCs and M2-like macrophages for NSCLC diagnosis and radiotherapy, we analyzed the percentages of MDSCs and M2-like macrophages between the HC group and the pre-RT group and found that the area under the curve (AUC) scores of the percentages of MDSCs and M2-like macrophages for NSCLC diagnosis were 0.930 (*p* = 0.001) and 0.711 (*p* = 0.098). In addition, we also analyzed the percentages of MDSCs and M2-like macrophages between the pre-RT group and the RT group, finding that AUC scores of the proportions of MDSCs and M2-like macrophages for NSCLC radiotherapy were 0.723 (*p* = 0.080) and 0.682 (*p* = 0.032) (Figures 4(a) and 4(b), Table 2–3). This part of data showed that MDSC was a more sensitive marker than CD68^+^CD163^+^M2-like macrophage for NSCLC diagnosis but CD68^+^CD163^+^M2-like macrophage could predict preferably whether the NSCLC patients have received radiotherapy.

### 3.6. The Effects of the Percentages of MDSCs and CD68^+^CD163^+^M2-Like Macrophages during Radiotherapy on the Therapeutic Response of NSCLC Patients

**Figure 5:**
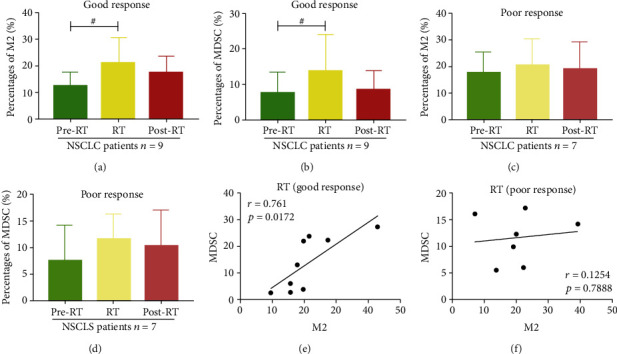
The therapeutic response role of the percentages of MDSCs and CD68^+^CD163^+^M2-like macrophages of NSCLC patients. (a, c) The percentages of CD68^+^CD163^+^M2-like macrophages of good and poor groups in pre-RT, RT, and post-RT. (b, d) The frequencies of MDSCs of good and poor in pre-RT, RT, and post-RT. (e, f) The correlation between MDSCs and CD68^+^CD163^+^M2-like macrophages of NSCLC patients of the good and poor groups during radiotherapy. ^#^*p* < 0.05. Data were analyzed by Pearson's correlation test. The bars denote mean ± SD.

After radiotherapy, we defined CR and PR as a good response group and SD and PD as a poor response group for NSCLC patients. We found that as for the NSCLC patients who reached a good response, the frequencies of MDSCs and CD68^+^CD163^+^M2-like macrophages in RT were elevated, in comparison with those for the pre-RT group (Figures 5(a) and 5(b)), while as for the NSCLC patients who reached a poor response, the percentages of two kinds of cells did not have an obvious alteration (Figures 5(c) and 5(d)). By the correlation analysis between MDSCs and CD68^+^CD163^+^M2-like macrophages of NSCLC patients in the good response or poor response group during radiotherapy, we found that these two kinds of peripheral cells of the good response group had an obvious and positive correlation, but the poor response group did not, demonstrating that ionizing radiation could simultaneously increase their percentages in peripheral blood and MDSCs and CD68^+^CD163^+^M2-like macrophages could act as biomarkers for treatment responses in NSCLC patients during radiotherapy (Figures 5(e) and 5(f)).

### 3.7. The Dynamic Changes of Blood Biochemical Indices and Their Correlation Analysis with M2-Like Macrophage and MDSCs in NSCLC Patients during Radiotherapy

**Figure 6:**
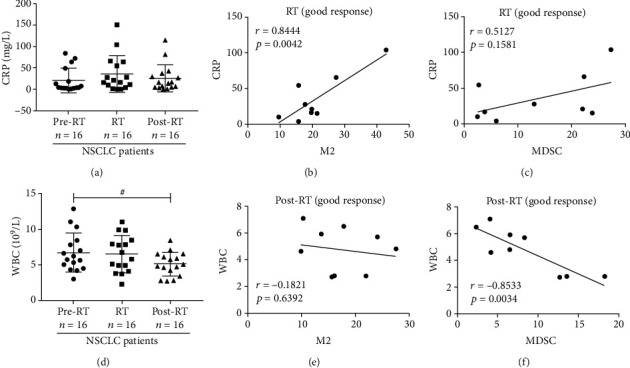
The crossed correlation analysis of CRP and WBCs with MDSCs and CD68^+^CD163^+^ M2-like macrophages in NSCLC patients. (a) The bar graph of the expressions of CRPs in NSCLC patients of pre-RT, RT, and post-RT groups. (b, c) The correlation of CRPs of the good response group with CD68^+^CD163^+^ M2-like macrophages and MDSCs of NSCLC patients during radiotherapy. (d) The plot of the expressions of WBCs in NSCLC patients of pre-RT, RT, and post-RT groups. (e, f) The correlation of WBCs of the good response group with CD68^+^CD163^+^M2-like macrophages and MDSCs of NSCLC patients after radiotherapy. ^#^*p* < 0.05. Data were analyzed by Pearson's correlation test.

We consulted the clinical data of patients and summarized their biochemical indicators, as shown in Table 4. We found that after radiotherapy, the expression levels of WBC, PLT, and direct bilirubin were decreased, indicating that radiotherapy can inhibit the bone marrow hematopoietic function. In addition, we further found that the expressions of C-reactive proteins (CRPs) of NSCLC patients were increased during radiotherapy and they were markedly and positively irrelated with the percentages of CD68^+^CD163^+^M2-like macrophages in the good response group (*r* = 0.8197, *p* = 0.0037) but did not have a significant relation with MDSCs (Figures 6(a)–6(c)). Our results also showed that the expressions of white blood cells (WBCs) of NSCLC patients were clearly decreased in the post-RT group, in comparison with the pre-RT group (Figure 6(d)). Moreover, the expressions of WBCs were significantly and negatively correlated with MDSCs in the good response group (*r* = −0.7934, *p* = 0.0062) but were not relevant to CD68^+^CD163^+^M2-like macrophages of NSCLC patients after radiotherapy (Figures 6(e) and 6(f)).

### 3.8. The Plasma Cytokines Were Examined by Multiplex ELISA in HC and NSCLC Patients

**Figure 7:**
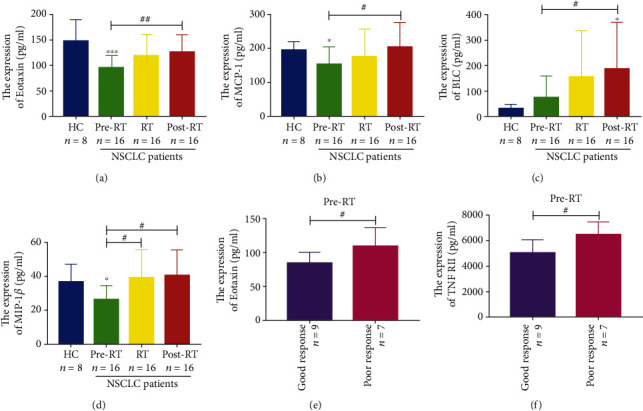
The expressions of plasma cytokines in HC and NSCLC patients. (a–d) The expressions of eotaxin, MCP-1, BLC, and MIP-1*β* in HC and NSCLC patients. (e, f) Histogram of the expressions of eotaxin and TNF RII in the good and poor response groups. ^∗^*p* < 0.05, ^∗∗^*p* < 0.01, ^∗∗∗^*p* < 0.001, and ^∗∗∗∗^*p* < 0.0001 compared with the HC group. ^#^*p* < 0.05 and ^##^*p* < 0.01. The bars denote mean ± SD.

Measuring 40 inflammation-associated cytokines of NSCLC patients and HC, as shown in Table 5, we found that 8 kinds of cytokines (BLC, eotaxin, I-309, MCP-1, MIP-1*β*, RANTES, TNF RI, and TNF RII) were significantly elevated in NSCLC patients, in comparison with HC. Especially, eotaxin, MCP-1, and MIP-1*β* were markedly decreased in NSCLC patients of the pre-RT group, in comparison with the HC group, but they and BLC were significantly elevated after radiotherapy compared with the pre-RT group (Figures 7(a)–7(d)). 17 kinds of plasma cytokines (G-CSF, GM-CSF, ICAM-1, IFN-*γ*, IL-1*α*, IL-2, IL-4, IL-5, IL-7, IL-8, IL-10, IL-12p70, IL-13, IL-15, IL-17, TNF-*α*, and TNF-*β*) were obviously decreased in NSCLC patients, in comparison with HC. However, 15 kinds of plasma cytokines (eotaxin-2, IL-1*β*, IL-1ra, IL-6, IL-6R, IL-11, IL-12p40, IL-16, MCSF, MIG, MIP-1*α*, MIP-1d, PDGF-BB, TIMP-1, and TIMP-2) hardly changed in NSCLC patients, in comparison with HC. This part of data in the table showed the dynamic alterations of plasma cytokines in NSCLC patients during radiotherapy. We further found that before radiotherapy, there were more high expression levels of eotaxin and TNF RII in the poor group than in the good group (Figures 7(e) and 7(f)), and the results indicated that the low expression levels of eotaxin and TNF RII could predict a better therapeutic response for NSCLC patients who had accepted ionizing radiation.

### 3.9. Correlation between the Expressions of Plasma Cytokines and MDSCs as well as CD68^+^CD163^+^M2-Like Macrophages of NSCLC Patients during Radiotherapy

**Figure 8:**
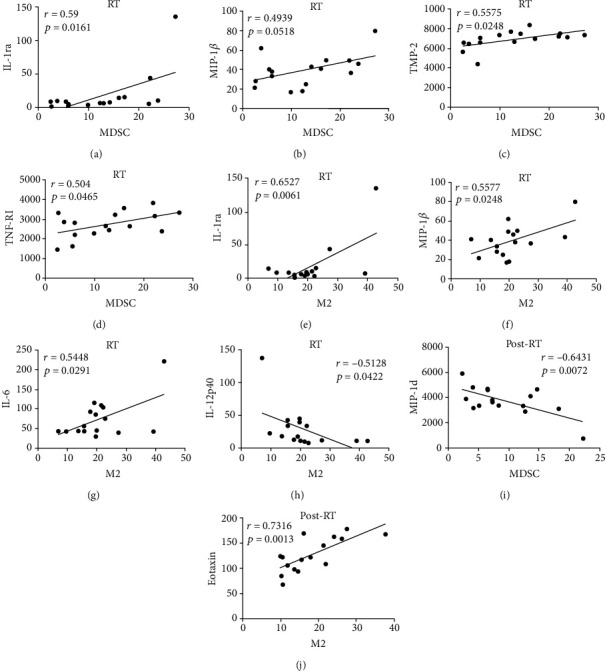
Correlation between the expressions of plasma cytokines and MDSCs as well as CD68^+^CD163^+^M2-like macrophages of NSCLC patients in RT and post-RT groups. (a–d) The expressions of MDSCs were correlated with IL-1ra, MIP-1*β*, TIMP-2, and TNF RI in NSCLC patients during radiotherapy. (e–h) The expressions of CD68^+^CD163^+^M2-like macrophages were correlated with IL-1ra, MIP-1*β*, IL-6, and IL-12p40 in NSCLC patients during radiotherapy. (i) Correlation between the expression of MDSCs and MIP-1d after radiotherapy. (j) Correlation between the expression of M2-like macrophage and eotaxin after radiotherapy. Data were analyzed by Pearson's correlation test.

Ionizing radiation increased the percentages of MDSCs and M2-like macrophages in NSCLC patients. However, the correlation between MDSCs and CD68^+^CD163^+^M2-like and plasma cytokines had barely been illuminated in NSCLC patients in ionizing radiation. In this study, as shown in Table S1, we explored their relationship and the indexes with significant correlation were shown in the scatter plots. We found that MDSCs were correlated positively with IL-1ra (*r* = 0.59, *p* = 0.0161), MIP-1*β* (*r* = 0.4939, *p* = 0.0516), TIMP-2 (*r* = 0.5575, *p* = 0.0248), and TNF RI (*r* = 0.504, *p* = 0.0465) in NSCLC patients of the RT group (Figures 8(a)–8(d)). CD68^+^CD163^+^M2-like macrophages were positively related with IL-1ra (*r* = 0.6527, *p* = 0.0061), MIP-1*β* (*r* = 0.5577, *p* = 0.0248), and IL-6 (*r* = 0.5448, *p* = 0.0291) in NSCLC patients of the RT group but were negatively related with IL-12p40 (*r* = −0.5128, *p* = 0.0422) (Figures 8(e)–8(h)). We also analyzed the relation of the expression levels of plasma cytokines with MDSCs and M2-like macrophage, finding that MDSCs were negatively related with MIP-1d (*r* = −0.6431, *p* = 0.0072) in NSCLC patients after radiotherapy (Figure 8(i)). The expression of CD68^+^CD163^+^M2-like macrophages was positively correlated with eotaxin (CCL11) (*r* = 0.7316, *p* = 0.0013) in NSCLC patients after radiotherapy (Figure 8(j)). This part of data indicated that the expressions of plasm cytokines could help to predict and increase the value of MDSCs and M2-like macrophages for NSCLC patients in radiotherapy.

## 4. Discussion

NSCLC is the leading pathology type of lung cancer [18]. Ionizing radiation therapy is a traditional method of treating advanced NSCLC patients [19]. As we all know, ionizing radiation (IR) is often regarded as an element of danger [20]. However, danger responses on the cellular and molecular level are often beneficial to the induction of antitumor immunity and amelioration of inflammation [21]. In the current study, through examining the percentages of MDSCs and CD68^+^CD163^+^M2-like macrophages, their relationships, their further changes in ionizing radiation therapy, and the cytokine profiles of NSCLC patients in radiotherapy, we provided novel insights into the role of myeloid-derived immune modulator cells in NSCLC patients during radiotherapy.

Myeloid-derived suppressor cells (MDSCs) are critical in regulating immune responses by suppressing antigen-presenting cells (APCs) and T cells [22]. In mice, MDSCs are usually defined as either CD11b^+^GR-1^+^ or CD11b^+^GR-1^−^ [23]. The surface markers of cells defining human MDSCs have not yet to be confirmed [24, 25], partly because no unified markers are currently available for these cells. However, these cells typically express the common myeloid markers, CD33 and CD11b, but lack markers of mature myeloid cells, such as HLA-DR [26]. Therefore, MDSCs are defined as CD33^+^CD11b^+^HLA-DR^−^ in NSCLC patients in this present study. Macrophages, key regulators of inhibition and resolution of inflammation [27], are a large group of immune cells, which can generally be divided into two subpopulations according to the specific immune responses involved, namely, M1 and M2 types [28, 29]. M2 macrophages that are usually identified based on the expression patterns of a set of diverse markers display anti-inflammatory properties and implicate in debris scavenging and tissue repair [29, 30]. CD163, a scavenger receptor expressed on cells of the monocyte lineage [31], was stained intracellularly in the present study to show the potential monocyte-to-M2 macrophage polarization. This cell population was defined as M2-like macrophages because only monocytes but not activated macrophages exist in the peripheral blood.

MDSC expansion is generally linked to inflammatory processes [32]. Recent studies showed that MDSCs could expand under pathological conditions including multiple sclerosis [33], 1 diabetes [34], rheumatoid arthritis [35], systemic lupus erythematosus [36], and autoimmune hepatitis [37]. Our study found that the expressions of MDSCs were markedly accumulated in NSCLC patients during radiotherapy, suggesting that MDSC might be involved in the peripheral immune response to radiotherapy. Although studies have shown multiple roles of MDSCs in the lung tumor microenvironment, including inhibition of tumor growth and progression mediated by antitumor immunity and the association of MDSCs with poor prognosis and increased resistance to chemotherapy and immunotherapy [38], the MDSCs in our study were free in the peripheral blood, not in the immune microenvironment, which might be play a vital role in anti-inflammation other than promoting cancer progression and metastasis. Therefore, we speculated that MDSCs might regulate the inflammation responses caused by ionizing radiation therapy, and the increasing MDSCs could be beneficial to improving the curative effects for advanced NSCLC patients. In addition, M2-like macrophages as an anti-inflammation ingredient were positively correlated with MDSCs in NSCLC patients during radiotherapy, which could further suppress the inflammation responses caused by ionizing radiation therapy. Usually, M2 or alternatively activated macrophages are activated by IL-4, IL-10, IL-13 [39], and glucocorticoid hormones, express high levels of IL-10 and low levels of IL-12 [40], and facilitate tumor progression [41]. Interestingly, plasm cytokines assay in our study showed that the expressions of IL-4, IL10, and IL-13 were significantly decreased in NSCLC patients, in comparison with HC, whereas the expressions of M2-like macrophages were obviously elevated. These evidences manifested that CD68^+^CD163^+^M2-like macrophages in peripheral blood were different from the alternatively activated M2 macrophages in issue or tumor microenvironment. Besides, although recent researches showed that the increasing MDSCs and M2 macrophages were markers for poor prognosis in cancer [42] due to promoting invasiveness [43] or tumor progression [44], MDSCs and CD68^+^CD163^+^M2-like macrophages in the present study only were provisionally elevated in NSCLC patients during radiotherapy. The frequencies of MDSCs and CD68^+^CD163^+^M2-like macrophages of NSCLC patients in the post-RT group returned to the level of NSCLC patients in the pre-RT group, which could illustrate that the percentages of MDSCs and CD68^+^CD163^+^M2 macrophages were transiently increased in NSCLC patients during radiotherapy due to the inflammation reaction caused by ionizing radiation, and CD68^+^CD163^+^M2 macrophages could be different from M2 or alternatively activated macrophages. However, whether the elevated MDSCs and CD68^+^CD163^+^M2-like macrophages promote tumor metastasis still needs a longer follow-up visit in NSCLC patients during radiotherapy and also deserves further investigations. In addition, we found that during radiotherapy, the proportions of MDSCs were markedly increased in non-small cell lung adenocarcinoma patients but not in non-small cell lung squamous carcinoma patients; however, the proportions of CD68^+^CD163^+^M2-like macrophages were markedly elevated in non-small cell lung squamous carcinoma patients but not in non-small cell lung adenocarcinoma patients. Hence, we predicted that during radiotherapy, alterations of the percentages of MDSCs are more sensitive in lung adenocarcinoma patients, while CD68^+^CD163^+^M2-like macrophages are more specific for lung squamous carcinoma patients. We also found that there were much more MDSCs and CD68^+^CD163^+^M2-like macrophages of NSCLC patients without metastasis in the RT and post-RT groups than in the pre-RT group. In comparison with NSCLC patients with metastasis, the percentages of MDSCs and M2-like macrophages were also decreased in NSCLC patients without metastasis in the pre-RT group. As for the NSCLC patients between the good and poor response groups, the former percentages of MDSCs and CD68^+^CD163^+^M2-like macrophages were elevated during radiotherapy and the expressions of the two kinds of immune cells existed in a significant and positive correlation, but the latter not. Furthermore, the results of ROC curve analysis showed that MDSC could be used as a sensitive marker for NSCLC patient's diagnosis and M2-like macrophage could predict whether NSCLC patients have received radiotherapy. Therefore, all data suggested that MDSCs and CD68^+^CD163^+^M2-like macrophages could be expected to be as sensitive markers for NSCLC patients in radiotherapy.

C-Reactive protein (CRP) is a pentameric protein synthesized by the liver, whose level rises in response to inflammation [45]. CRP has been determined as a prognostic factor in nasopharyngeal carcinoma [46] and colon cancer [47]. In the present study, we found that the expression levels of CRP of NSCLC patients in the good response group during radiotherapy correlated positively with CD68^+^CD163^+^M2-like macrophages. The increasing levels of CRP further indicated that ionizing radiation therapy induced the inflammation responses, which could be a marker of therapeutic response for NSCLC patients in radiotherapy. We also found that WBCs were decreased after ionizing radiation therapy and WBCs were correlated significantly and negatively with MDSCs of NSCLC patients in the good response group after radiotherapy. Therefore, we speculated that the expressions of CRP and WBCs could help the MDSCs and CD68^+^CD163^+^M2-like macrophages to further predict the therapeutic response of NSCLC patients after radiotherapy.

Eotaxin (CCL11) has been shown to promote the chemotaxis of eosinophils cells by binding to chemokine receptor 3 [41], but its effects on CD68^+^CD163^+^M2-like macrophage recruitment remain unknown. Our data indicated that before radiotherapy, the low expression level of eotaxin was conducive to the therapeutic response to radiotherapy and the expression of eotaxin was positively correlated with CD68^+^CD163^+^M2-like macrophages of NSCLC patients after radiotherapy, suggesting that eotaxin could emerge as a potential marker for the prediction of radiotherapy efficacy and recruitment of CD68^+^CD163^+^M2-like macrophages of NSCLC patients in radiotherapy. MIP-1*β* (CCL4), RANTES, and the human cytokine I-309 are all monocyte and lymphocyte chemoattractant [48]. Our results showed that MIP-1*β* was obviously elevated in NSCLC patients after radiotherapy, in comparison with these patients before radiotherapy, and there were more expressions of RANTES and I-309 of NSCLC patients in the post-RT group than in the HC group. In the present study, MDSCs and CD68^+^CD163^+^M2-like macrophages of PBMCs were detected by flow cytometry and were visibly raised in NSCLC patients. We found that the expressions of MDSCs as well as CD68^+^CD163^+^M2-like macrophages were also positively correlated with MIP-1*β* in NSCLC patients during radiotherapy. This data indicated that MIP-1*β* could also recruit MDSCs and CD68^+^CD163^+^M2-like macrophages of NSCLC patients during radiotherapy, thereby playing a vital role in evaluating the inflammation environment of radiation therapy. In addition, we detected that the interleukin-1 receptor antagonist (IL-1ra) had a positive relationship with MDSCs and CD68^+^CD163^+^M2-like macrophages but the tissue inhibitor of metalloproteinases-2 (TIMP-2) and tumor necrosis factor receptor-I (TNF RI) were only correlated with MDSCs in NSCLC patients during radiotherapy. CD68^+^CD163^+^M2-like macrophages were negatively correlated with IL-12p40 but had a positive linear relation with IL-6. Some studies found that IL-1ra was a competitive inhibitor of IL-1(*α*/*β*) [49] and was neuroprotective in the mice that had a stroke [50]. IL-1ra could also suppress the growth of esophageal cancer cells by blocking IL-1*α* [51]. In this article, IL-1ra could play a crucial role because it was clearly related with MDSCs and CD68^+^CD163^+^M2-like macrophages in NSCLC patients during radiotherapy, which deserved deep investigations in the following experiments.

In ionizing radiation treatment, the percentages of MDSCs and CD68^+^CD163^+^M2-like macrophages were clearly elevated in NSCLC patients. We speculated that the increase of MDSCs and CD68^+^CD163^+^M2-like macrophages could be a reactive mechanism that suppressed the inflammation response caused by ionizing radiation. However, we still need to increase the number of NSCLC patients for further investigations, and if necessary, we will also explore the molecular mechanism in detail.

## 5. Conclusion

In conclusion, radiotherapy upregulated the expression of peripheric MDSCs and CD68^+^CD163^+^M2-like macrophages. The accumulations of circulating MDSCs and CD68^+^CD163^+^M2-like macrophages were found to be correlated with the good curative effect responses and the expressions of plasma cytokines and contributed to diagnosing NSCLC. MDSCs were positively correlated with CD68^+^CD163^+^M2-like macrophages in NSCLC patients during radiotherapy, indicating that upregulating them could provide auxiliary and sensitive biomarkers for NSCLC diagnosis and predict a better therapeutic response of NSCLC patients in radiotherapy.

## Figures and Tables

**Table 1:** Clinical characteristic of NSCLC patients in different stages of radiotherapy.

Characteristic	NSCLC patients in pre-RT		NSCLC patients in RT		NSCLC patients in post-RT	
	No.	%	No.	%	No.	%
Gender						
Male	6	37.5	6	37.5	6	37.5
Female	10	62.5	10	62.5	10	62.5
Ages, years, median (range)	72 (44, 83)	—	72 (44, 83)	—	72 (44, 83)	—
Histology						
Adenocarcinoma	10	62.5	10	62.5	10	62.5
Squamous	6	37.5	6	37.5	6	37.5
Stage						
III	4	25	4	25	4	25
IV	12	75	12	75	12	75
Radiotherapy area						
Lung	7	43.75	7	43.75	7	43.75
Brain	5	31.25	5	31.25	5	31.25
Bone	4	25	4	25	4	25
Response to radiotherapy						
Good response	9	56.25	9	56.25	9	56.25
Poor response	7	43.75	7	43.75	7	43.75

**Table 2:** ROC curve analysis about the diagnosis of NSCLC patients (area under the curve).

Test result variable(s)	Area	Std. error^a^	Asymptotic sig.^b^	Asymptotic 95% confidence interval	
				Lower bound	Upper bound
M2	0.711	0.123	0.098	0.469	0.953
MDSC	0.930	0.055	0.001	0.822	1.000

^a^Under the nonparametric assumption. ^b^Null hypothesis: true area = 0.5.

**Table 3:** ROC curve analysis about the radiotherapy of NSCLC patients (area under the curve).

Test result variable(s)	Area	Std. error^a^	Asymptotic sig.^b^	Asymptotic 95% confidence interval	
				Lower bound	Upper bound
M2	0.723	0.092	0.032	0.542	0.903
MDSC	0.682	0.096	0.080	0.494	0.869

^a^Under the nonparametric assumption. ^b^Null hypothesis: true area = 0.5.

**Table 4:** Results of blood biochemical indices in NSCLC patients at different stages of radiotherapy.

	Pre-RT (*n* = 16)	RT (*n* = 16)	Post-RT (*n* = 16)
CRP	20.78 (0.3–83.99)	35.88 (0.5–150.865)	25.57 (0.35–114.43)
WBC	6.73 (3–12.8)	6.49 (2.3–11)	5.08 (2.73–8.4)^#^
Neutrophil (×10^9^)	7.48 (2.28–42.85)	7.18 (1.68–38.45)	5.5 (1.67–25.19)
PLT (×10^9^)	257 (146–394)	220.56 (130–412)	186.13 (79–332)^##^
Hb (g/l)	127.44 (87–183)	123.03 (84–153)	119.31 (65–147)
Lymphocyte (×10^9^)	1.18 (0.5–2.59)	1.03 (0.42–1.88)	0.91 (0.28–2.54)
ALT (U/l)	20.25 (7–50)	48.53 (4–225)	40.31 (4–163)
AST (U/l)	32.88 (15–158)	58.09 (13–216)	53.5 (9–160)
GGT (U/l)	77.5 (16–367)	135.97 (13–660)	157.25 (14–863)
ALP (U/l)	124.13 (52–239)	138.84 (38–242)	122.69 (37–198)
Albumin (g/l)	35.5 (27–48)	34.16 (29–40)	32.53 (22–45)
Globulin (g/l)	33.63 (23–55)	33.03 (26–43)	34.07 (27–47)
Tbil (*μ*mol/l)	11.21 (3–25.8)	8.26 (3–12.3)	9.71 (5–24)
Direct bilirubin (*μ*mol/l)	1.93 (0–3.8)	0.38 (0–2.6)^∗∗^	0.41 (0–3)^###^
Creatinine (*μ*mol/l)	68.56 (42–96)	68.59 (42–99)	68.88 (36–90)
UA (*μ*mol/l)	317.13 (192–508)	290.84 (169–466)	274.31 (107–551)
Carbamide (mmol/l)	5.54 (3.4–9.8)	6.25 (2.5–12)	5.83 (2.8–10)

Data are presented as mean (interquartile range). pre-RT: preradiotherapy; RT: radiotherapy; post-RT: postradiotherapy; WBC: white blood cell; CRP: C-reactive protein; PLT: platelet; Hb: hemoglobin; ASL: glutamic oxalacetic transaminase; ALT: glutamic-pyruvic transaminase; GGT: gamma-glutamyl transferase; ALP: alkaline phosphatase; Tbil: total bilirubin; UA: uric acid; pre-RT vs RT, ^∗^*p* < 0.05, ^∗∗^*p* < 0.01, and ^∗∗∗^*p* < 0.001; pre-RT vs post-RT, ^#^*p* < 0.05, ^##^*p* < 0.01, and ^###^*p* < 0.001.

**Table 5:** Results of plasma cytokine assay in healthy controls and NSCLC patients (pg/ml).

	Health controls (*n* = 8)	NSCLC patients		
		Pre-RT (*n* = 16)	RT (*n* = 16)	Post-RT (*n* = 16)
BLC	27.41 (11.61–69.61)	73.63 (12.87–286.32)	156.24 (16.72–666.31)	186.29 (20.43–514.35)^∗^
Eotaxin	147.89 (109.28–228.22)	94.84 (56.68–137.3)^∗∗∗^	118.44 (54.95–177.57)	126.52 (67.84–177.98)
Eotaxin-2	127.28 (70.63–187.24)	110.01 (11.28–262.55)	133.25 (42.01–265.49)	130.79 (53.21–264.85)
G-CSF	46.73 (3.95–184.06)	64.04 (0–905.69)^∗^	64.63 (0.25–584.34)^∗^	21.62 (4.04–139.05)
GM-CSF	200.87 (114.71–285.43)	136.91 (64.75–236.43)^∗^	125.27 (50.87–272.29)^∗∗^	107.9 (39.27–273.09)^∗∗^
I-309	0.72 (0–4.21)	25.24 (0–331.4)^∗∗^	13.03 (0–130.58)^∗∗^	10.02 (0–51.88)^∗^
ICAM-1 (×10^3^)	20.98 (18.54–24.28)	17.45 (7.5–23.10)^∗^	17.61 (11.31–21.59)^∗^	18.26 (11.50–23.91)
IFN-*γ*	300.64 (185.68–424.81)	247.44 (117.81–420.07)	217.9 (89.21–491.77)	197.61 (109.57–469.69)^∗^
IL-1*α*	33.94 (20.36–46.56)	11.3 (1.91–31.71)^∗∗∗∗^	8.03 (0.78–24.01)^∗∗∗∗^	6.62 (0–23.6)^∗∗∗∗^
IL-1*β*	1.11 (0–8.91)	9.59 (0–150.85)	4.34 (0–67.69)	0.98 (0–14.91)
IL-1ra	5.48 (4.04–7.53)	14.63 (2.54–55.81)	17.93 (1.67–135.67)	14.85 (1.23–54.23)
IL-2	19.15 (10.29–32.66)	12.32 (5.17–22.37)^∗^	10.43 (4.11–21.41)^∗∗^	9.25 (3.66–18.12)^∗∗∗^
IL-4	81.06 (45.89–127.04)	32.42 (2.22–117.83)^∗∗^	22.87 (0–64.6)^∗∗∗∗^	19.49 (0.05–59.9)^∗∗∗∗^
IL-5	61.58 (41.33–89.77)	38.27 (17.96–66.01)^∗∗^	34.44 (14.01–84.83)^∗∗^	31.45 (9.66–65.89)^∗∗∗^
IL-6	83.3 (56.92–115.93)	81.52 (27.31–153.12)	74.87 (30.47–222.07)	66.91 (33.25–95.56)
IL-6R (×10^3^)	2.46 (2.02–3.00)	2.5 (1.71–3.16)	2.43 (1.86–3.10)	2.44 (2.10–2.78)
IL-7	114.55 (77.97–169.47)	72.2 (40.25–117.13)^∗∗∗^	67.55 (33.92–116.7)^∗∗^	62.42 (27.52–97.6)^∗∗∗∗^
IL-8	46.16 (29.58–63.42)	39.08 (13.19–83.77)	33.66 (9.6–69.15)	29.44 (3.82–61.95)^∗^
IL-10	28.34 (18.32–39.12)	21.05 (7.45–37.27)	20.29 (9.56–42.14)	17.56 (8.85–39.11)^∗∗^
IL-11 (×10^3^)	0.52 (0.32–0.63)	0.53 (0.21–1.87)	0.48 (0.17–1.75)	0.50 (0.22–1.67)
IL-12p40	46.68 (12.53–86.01)	52.94 (7.45–292.73)	28.93 (7.56–137.21)	50.36 (3.38–236.59)
IL-12p70	3.63 (2.41–5.29)	2.99 (0.73–5.3)	2.95 (1.63–5.27)	2.16 (0.68–4.8)^∗∗^
IL-13	80.72 (54.01–110.56)	51.15 (18.9–98.46)^∗∗^	48.23 (19.27–90.37)^∗∗^	41.76 (13.49–89.51)^∗∗∗^
IL-15	157.97 (94.63–205.65)	128.07 (67.21–234.93)	107.08 (0–200.38)	95.17 (30.29–202.59)
IL-16	28.6 (15.19–38.37)	58.11 (7.4–380.9)	28.74 (5.59–76.82)	20.62 (9.09–45.19)
IL-17	10.54 (6.36–18.56)	8.01 (0.91–38.4)	5.52 (0.28–35.99)	4.5 (0.06–27.53)^∗^
MCP-1	195.82 (171.53–230.02)	153.29 (75.72–270.93)^∗^	174.08 (53.52–378.28)	203.18 (89.98–342)
MCSF	0 (0–0)	1.05 (0–13.67)	0.53 (0–3.32)	0.2 (0–0.99)
MIG	1.03 (0–5.64)	44.07 (0–499.69)	43.09 (0–334.09)	109.6 (0–669.56)
MIP-1*α*	59.88 (25.68–182.29)	76.9 (14.56–296.98)	85.26 (27.62–392.83)	80.63 (30.64–181.63)
MIP-1*β*	36.54 (16.33–50.45)	26.32 (13.74–41.69)^∗^	39.11 (16.59–79.61)	40.58 (17.45–69.73)
MIP-1d (×10^3^)	4.10 (2.53–5.26)	3.68 (2.36–5.29)	3.46 (2.00–5.72)	3.75 (0.72–5.92)
PDGF-BB	479.88 (392.19–539.18)	483.86 (343.26–619.91)	447.45 (326.7–573.83)	433.7 (215.86–585.86)
RANTES (×10^3^)	1.98 (1.72–2.42)	2.06 (1.62–2.60)	2.12 (1.70–2.63)	2.17 (1.91–2.60)^∗^
TIMP-1 (×10^3^)	5.02 (4.54–5.57)	5.03 (3.96–6.06)	5.09 (3.97–5.82)	5.10 (3.82–5.73)
TIMP-2 (×10^3^)	7.11 (5.38–8.50)	7.11 (5.94–9.23)	6.90 (4.38–8.36)	7.35 (6.37–8.76)
TNF-*α*	794.8 (326.76–1100.16)	415.58 (132.32–734.35)^∗∗∗^	394.79 (121.71–963.41)^∗∗^	326.07 (97.02–698.34)^∗∗∗^
TNF-*β* (×103)	0.66 (0.30–0.95)	0.43 (0.065–1.59)	0.32 (0.068–0.92)^∗∗^	0.32 (0–1.03)^∗∗^
TNF RI (×10^3^)	1.78 (0.66–2.49)	2.68 (1.37–5.28)^∗^	2.75 (1.48–3.84)^∗∗^	2.82 (1.79–3.89)^∗∗∗^
TNF RII (×10^3^)	4.25 (3.45–5.45)	5.64 (3.71–8.24)^∗∗^	5.83 (4.41–8.09)^∗∗∗^	6.20 (4.39–8.32)^∗∗∗^

Data are presented as mean (interquartile range). CCL11: CeC motif chemokine 11; G-CSF: granulocyte colony-stimulating factor; GM-CSF: granulocyte–macrophage colony-stimulating factor; ICAM-1: intercellular adhesion molecule-1; IFN-*γ*: interferon-gamma; IL: interleukin; MCP-1: monocyte chemoattractant protein-1; M-CSF: macrophage colony-stimulating factor; CXCL9: CXC ligand 9; MIP: macrophage-inflammatory protein; PDGF-BB: platelet-derived growth factor BB; RANTES: regulated on activation normal T expressed and secreted chemokines; TIMP: tissue inhibitor of metalloproteinases; TNF: tumor necrosis factor; TNFR: tumor necrosis factor receptor. ^∗^*p* < 0.05, ^∗∗^*p* < 0.01, ^∗∗∗^*p* < 0.001, and ^∗∗∗∗^*p* < 0.0001 compared with healthy controls.

## Data Availability

The data in this study is available from the corresponding author upon reasonable request.

## Abbreviations

NSCLC: Non-small cell lung cancer
MDSCs: Myeloid-derived suppressor cells
DCs: Dendritic cells
pre-RT: Preradiotherapy
RT: Radiotherapy
post-RT: Postradiotherapy
CR: Complete response
PR: Partial response
PD: Progressive disease
SD: Stable disease
HC: Healthy control
IR: Ionizing radiation
CRP: C-Reactive protein
WBCs: White blood cells
APCs: Antigen-presenting cells
CCL11: CeC motif chemokine 11
G-CSF: Granulocyte colony-stimulating factor
GM-CSF: Granulocyte–macrophage colony-stimulating factor
ICAM-1: Intercellular adhesion molecule-1
IFN-*γ*: Interferon-gamma
IL: Interleukin
MCP-1: Monocyte chemoattractant protein-1
M-CSF: Macrophage colony-stimulating factor
CXCL9: CXC ligand 9
MIP: Macrophage-inflammatory protein
PDGF-BB: Platelet-derived growth factor BB
RANTES: Regulated on activation normal T expressed and secreted chemokines
TIMP: Tissue inhibitor of metalloproteinases
TNF: Tumor necrosis factor
TNFR: Tumor necrosis factor receptor.

## Supplementary Materials

Supplementary Materials Table S1: correlation analyses between M2 with MDSC and plasma cytokines at different stages of radiotherapy for NSCLC patients. Figure S1: the FSC × SSC gating strategy of PBMCs for gating MDSCs (A) and M2-like macrophages (B).
