# Supplementary material for: Myeloid-Derived Suppressor Cells and CD68+CD163+M2-Like Macrophages as Therapeutic Response Biomarkers Are Associated with Plasma Inflammatory Cytokines: A Preliminary Study for Non-Small Cell Lung Cancer Patients in Radiotherapy
Source: J Immunol Res. 2022 Jul 26;2022:3621496. doi: 10.1155/2022/3621496 (PMC9345704; doi:10.1155/2022/3621496)
Supplement: Supplementary Materials — Table S1: correlation analyses between M2 with MDSC and plasma cytokines at different stages of radiotherapy for NSCLC patients. Figure S1: the FSC × SSC gating strategy of PBMCs for gating MDSCs (A) and M2-like macrophages (B). [file 3621496.f1.docx]

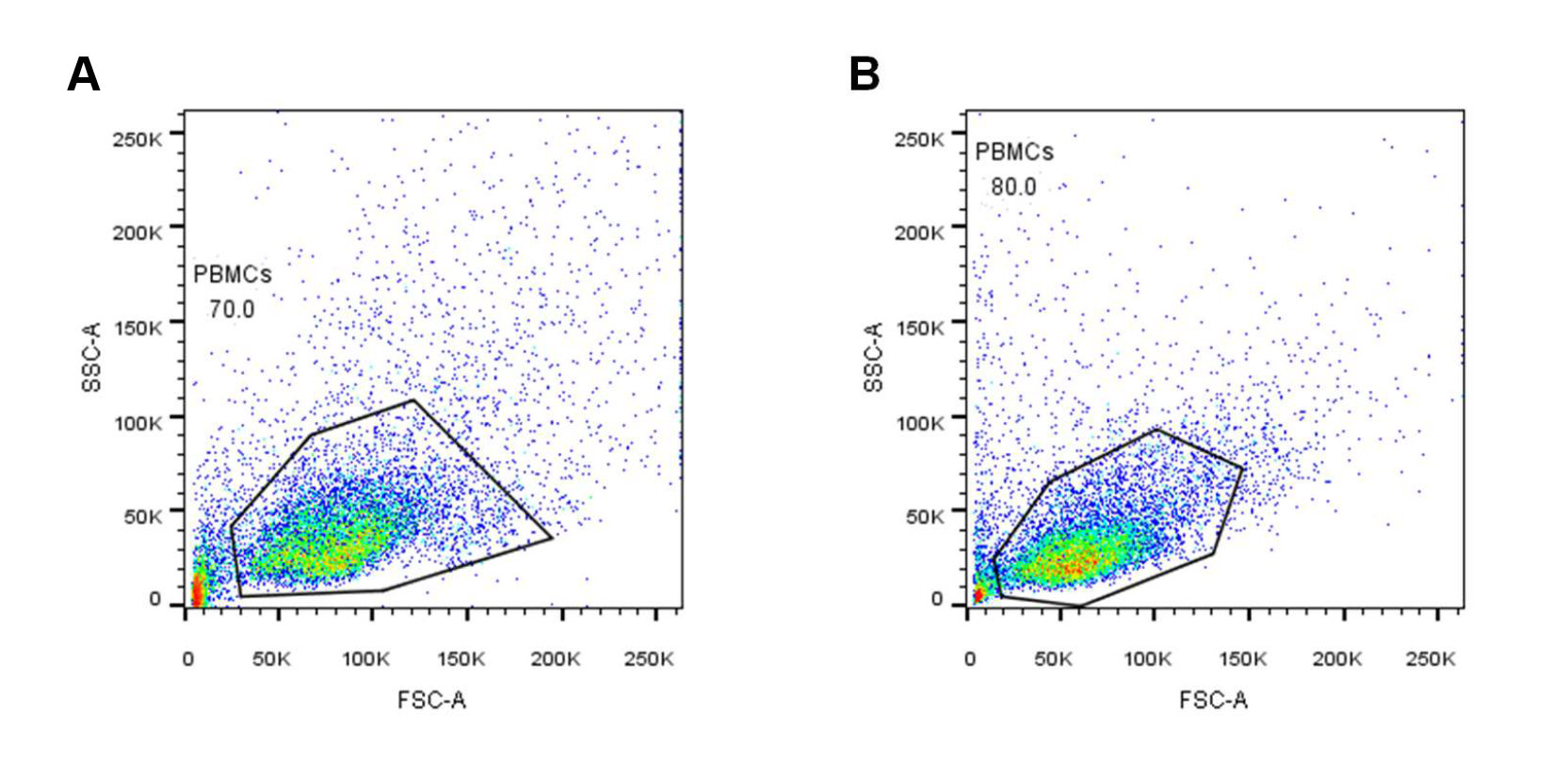


**Figure S1** The FSC × SSC gating strategy of PBMCs for gating MDSCs **(A)** and M2-like macrophages **(B)**

| **Table S1 Correlation analyses between M2 with MDSC and Plasma cytokines at different stages of radiotherapy for NSCLC patients** | | | | | | | | | | | | | | |  |  |
| --- | --- | --- | --- | --- | --- | --- | --- | --- | --- | --- | --- | --- | --- | --- | --- | --- |
|  |  |  |  |  |  |  |  |  |  |  |  |  |  |  |  | |
| **pre-RT** | **M2** | | **MDSC** | | **RT** | **M2** | | **MDSC** | | **post-RT** | **M2** | | **MDSC** | |  |  |
|  | **Cor** | **p** | **Cor** | **p** |  | **Cor** | **p** | **Cor** | **p** |  | **Cor** | **p** | **Cor** | **p** |  |  |
| BLC | -0.136 | 0.615 | 0.125 | 0.644 | BLC | -0.027 | 0.920 | 0.451 | 0.080 | BLC | -0.090 | 0.740 | 0.002 | 0.993 |  |  |
| Eotaxin | -0.048 | 0.860 | 0.230 | 0.392 | Eotaxin | -0.091 | 0.737 | -0.301 | 0.258 | Eotaxin | .732^**^ | 0.001 | 0.407 | 0.118 |  |  |
| Eotaxin-2 | -0.280 | 0.294 | 0.298 | 0.262 | Eotaxin-2 | -0.266 | 0.319 | -0.115 | 0.673 | Eotaxin-2 | 0.378 | 0.149 | -0.114 | 0.676 |  |  |
| G-CSF | -0.329 | 0.214 | -0.086 | 0.753 | G-CSF | 0.348 | 0.187 | .497^*^ | 0.050 | G-CSF | -0.351 | 0.183 | -0.263 | 0.325 |  |  |
| GM-CSF | 0.095 | 0.726 | 0.016 | 0.953 | GM-CSF | -0.121 | 0.655 | -0.149 | 0.583 | GM-CSF | 0.123 | 0.650 | -0.022 | 0.936 |  |  |
| I-309 | -0.304 | 0.252 | -0.068 | 0.802 | I-309 | -0.352 | 0.182 | 0.131 | 0.629 | I-309 | -0.179 | 0.508 | 0.119 | 0.660 |  |  |
| ICAM-1 | -0.382 | 0.144 | -0.041 | 0.881 | ICAM-1 | -0.020 | 0.941 | -0.104 | 0.701 | ICAM-1 | 0.112 | 0.680 | -0.021 | 0.939 |  |  |
| IFNγ | -0.045 | 0.868 | 0.033 | 0.905 | IFNγ | -0.224 | 0.404 | -0.102 | 0.706 | IFNγ | 0.061 | 0.824 | -0.107 | 0.692 |  |  |
| IL-1α | -0.156 | 0.564 | -0.327 | 0.217 | IL-1α | -0.221 | 0.411 | -0.111 | 0.682 | IL-1α | 0.099 | 0.715 | 0.005 | 0.987 |  |  |
| IL-1β | -0.316 | 0.233 | -0.068 | 0.804 | IL-1β | -0.397 | 0.128 | 0.112 | 0.679 | IL-1β | -0.244 | 0.362 | -0.083 | 0.759 |  |  |
| IL-1ra | -0.293 | 0.271 | -0.185 | 0.493 | IL-1ra | .653^**^ | 0.006 | .590^*^ | 0.016 | IL-1ra | -0.370 | 0.158 | -0.298 | 0.262 |  |  |
| IL-2 | 0.216 | 0.422 | -0.075 | 0.784 | IL-2 | -0.137 | 0.613 | -0.053 | 0.844 | IL-2 | 0.246 | 0.358 | -0.008 | 0.977 |  |  |
| IL-4 | -0.196 | 0.467 | -0.231 | 0.389 | IL-4 | -0.167 | 0.537 | 0.030 | 0.913 | IL-4 | 0.074 | 0.785 | -0.077 | 0.776 |  |  |
| IL-5 | 0.072 | 0.790 | 0.024 | 0.929 | IL-5 | -0.154 | 0.570 | -0.092 | 0.734 | IL-5 | 0.268 | 0.316 | -0.046 | 0.865 |  |  |
| IL-6 | 0.477 | 0.062 | -0.171 | 0.527 | IL-6 | .545^*^ | 0.029 | 0.371 | 0.157 | IL-6 | -0.058 | 0.831 | -0.167 | 0.535 |  |  |
| IL-6R | -0.309 | 0.244 | -0.252 | 0.346 | IL-6R | 0.050 | 0.853 | 0.343 | 0.193 | IL-6R | -0.175 | 0.517 | -0.380 | 0.147 |  |  |
| IL-7 | 0.201 | 0.456 | -0.058 | 0.830 | IL-7 | -0.112 | 0.680 | -0.163 | 0.547 | IL-7 | 0.261 | 0.328 | 0.071 | 0.793 |  |  |
| IL-8 | 0.325 | 0.219 | -0.059 | 0.827 | IL-8 | 0.024 | 0.931 | -0.088 | 0.746 | IL-8 | 0.234 | 0.382 | 0.138 | 0.611 |  |  |
| IL-10 | 0.162 | 0.550 | -0.113 | 0.676 | IL-10 | -0.062 | 0.821 | -0.082 | 0.763 | IL-10 | -0.059 | 0.828 | -0.013 | 0.963 |  |  |
| IL-11 | -0.095 | 0.727 | -0.270 | 0.312 | IL-11 | -0.210 | 0.434 | -0.406 | 0.119 | IL-11 | -0.147 | 0.587 | -0.407 | 0.118 |  |  |
| IL-12p40 | -0.239 | 0.372 | -0.077 | 0.778 | IL-12p40 | -.513^*^ | 0.042 | -0.093 | 0.731 | IL-12p40 | 0.015 | 0.957 | 0.379 | 0.147 |  |  |
| IL-12p70 | -0.141 | 0.602 | -0.055 | 0.840 | IL-12p70 | 0.009 | 0.972 | 0.022 | 0.937 | IL-12p70 | 0.155 | 0.568 | 0.025 | 0.926 |  |  |
| IL-13 | 0.056 | 0.836 | -0.039 | 0.887 | IL-13 | -0.167 | 0.536 | -0.154 | 0.570 | IL-13 | 0.062 | 0.819 | -0.089 | 0.744 |  |  |
| IL-15 | 0.043 | 0.874 | -0.095 | 0.725 | IL-15 | 0.053 | 0.844 | 0.161 | 0.552 | IL-15 | -0.096 | 0.722 | -0.150 | 0.580 |  |  |
| IL-16 | -0.297 | 0.263 | -0.113 | 0.677 | IL-16 | 0.380 | 0.146 | 0.415 | 0.110 | IL-16 | -0.113 | 0.676 | 0.134 | 0.620 |  |  |
| IL-17 | -0.084 | 0.758 | -0.407 | 0.118 | IL-17 | -0.317 | 0.232 | -0.457 | 0.075 | IL-17 | -0.089 | 0.743 | -0.441 | 0.087 |  |  |
| MCP-1 | 0.172 | 0.523 | 0.012 | 0.965 | MCP-1 | 0.261 | 0.328 | 0.418 | 0.107 | MCP-1 | -0.238 | 0.375 | -0.022 | 0.937 |  |  |
| MCSF | -0.280 | 0.293 | 0.071 | 0.793 | MCSF | 0.185 | 0.493 | 0.001 | 0.996 | MCSF | 0.101 | 0.710 | -0.393 | 0.132 |  |  |
| MIG | -0.273 | 0.306 | -0.097 | 0.719 | MIG | -0.288 | 0.280 | 0.141 | 0.602 | MIG | 0.001 | 0.996 | 0.348 | 0.187 |  |  |
| MIP-1α | -0.340 | 0.197 | -0.190 | 0.482 | MIP-1α | -0.158 | 0.559 | -0.179 | 0.506 | MIP-1α | -0.175 | 0.517 | -0.289 | 0.277 |  |  |
| MIP-1β | 0.156 | 0.565 | 0.185 | 0.493 | MIP-1β | .558^*^ | 0.025 | 0.494 | 0.052 | MIP-1β | 0.198 | 0.463 | 0.102 | 0.706 |  |  |
| MIP-1d | -0.365 | 0.164 | -0.344 | 0.192 | MIP-1d | -0.314 | 0.236 | -0.147 | 0.586 | MIP-1d | -0.209 | 0.438 | -.643^**^ | 0.007 |  |  |
| PDGF-BB | 0.431 | 0.096 | 0.141 | 0.602 | PDGF-BB | 0.156 | 0.564 | -0.111 | 0.682 | PDGF-BB | 0.304 | 0.253 | -0.023 | 0.931 |  |  |
| RANTES | -0.488 | 0.055 | 0.080 | 0.769 | RANTES | 0.116 | 0.669 | 0.157 | 0.561 | RANTES | 0.000 | 1.000 | -0.423 | 0.103 |  |  |
| TIMP-1 | 0.011 | 0.969 | 0.164 | 0.544 | TIMP-1 | -0.455 | 0.077 | -0.383 | 0.143 | TIMP-1 | 0.189 | 0.484 | -0.269 | 0.313 |  |  |
| TIMP-2 | -0.100 | 0.713 | 0.272 | 0.309 | TIMP-2 | 0.283 | 0.288 | .558^*^ | 0.025 | TIMP-2 | -0.481 | 0.059 | -0.058 | 0.832 |  |  |
| TNFα | 0.180 | 0.504 | -0.187 | 0.488 | TNFα | -0.223 | 0.405 | -0.281 | 0.293 | TNFα | 0.159 | 0.557 | 0.016 | 0.954 |  |  |
| TNFβ | 0.155 | 0.567 | -0.090 | 0.741 | TNFβ | -0.177 | 0.511 | -0.180 | 0.504 | TNFβ | -0.137 | 0.614 | -0.152 | 0.573 |  |  |
| TNF RI | -0.128 | 0.637 | -0.040 | 0.884 | TNF RI | 0.386 | 0.140 | .504^*^ | 0.047 | TNF RI | -0.025 | 0.928 | -0.081 | 0.767 |  |  |
| TNF RII | 0.173 | 0.522 | -0.113 | 0.676 | TNF RII | 0.104 | 0.702 | 0.366 | 0.163 | TNF RII | -0.452 | 0.078 | -0.261 | 0.330 |  |  |
| **. At 0.01 level (two-tailed), the correlation was significant. | | | | | | | |  |  |  |  |  |  |  |  |  |
| *. At 0.05 level (two-tailed), the correlation was significant. | | | | | | | |  |  |  |  |  |  |  |  |  |
| Data were analyzed by Pearson’s correlation test. CCL11: CeC motif chemokine 11; G-CSF: granulocyte colony-stimulating factor; GM-CSF: granulocyte–macrophage colony-stimulating factor; ICAM-1: intercellular adhesion molecule-1; IFN-γ: interferon-gamma; IL: interleukin; MCP-1: monocyte chemoattractant protein-1; M-CSF: macrophage colony-stimulating factor; CXCL9: CXC ligand 9; MIP: macrophage-inflammatory protein; PDGF-BB: platelet-derived growth factor BB; RANTES: regulated on activation normal T expressed and secreted chemokines; TIMP: tissue inhibitor of metalloproteinases; TNF: tumor necrosis factor; TNFR: tumor necrosis factor receptor. | | | | | | | | | | | | | | |  | |
|  |  |  |  |  |  |  |  |  |  |  |  |  |  |  |  | |
|  |  |  |  |  |  |  |  |  |  |  |  |  |  |  |  | |
|  |  |  |  |  |  |  |  |  |  |  |  |  |  |  |  | |
|  |  |  |  |  |  |  |  |  |  |  |  |  |  |  |  | |
|  |  |  |  |  |  |  |  |  |  |  |  |  |  |  |  | |
